# Comparison of Modern Highly Interactive Flicker-Free Steady State Motion Visual Evoked Potentials for Practical Brain–Computer Interfaces

**DOI:** 10.3390/brainsci10100686

**Published:** 2020-09-28

**Authors:** Piotr Stawicki, Ivan Volosyak

**Affiliations:** Faculty of Technology and Bionics, Rhine-Waal University of Applied Sciences, 47533 Kleve, Germany; piotr.stawicki@hochschule-rhein-waal.de

**Keywords:** brain–computer interface (BCI), steady-state motion visual evoked potentials (SSMVEP), steady-state visual evoked potentials (SSVEP), flicker-free steady-state motion visual evoked potentials (FFSSMVEP), motion visual evoked potentials (mVEP)

## Abstract

Motion-based visual evoked potentials (mVEP) is a new emerging trend in the field of steady-state visual evoked potentials (SSVEP)-based brain–computer interfaces (BCI). In this paper, we introduce different movement-based stimulus patterns (steady-state motion visual evoked potentials—SSMVEP), without employing the typical flickering. The tested movement patterns for the visual stimuli included a pendulum-like movement, a flipping illusion, a checkerboard pulsation, checkerboard inverse arc pulsations, and reverse arc rotations, all with a spelling task consisting of 18 trials. In an online experiment with nine participants, the movement-based BCI systems were evaluated with an online four-target BCI-speller, in which each letter may be selected in three steps (three trials). For classification, the minimum energy combination and a filter bank approach were used. The following frequencies were utilized: 7.06 Hz, 7.50 Hz, 8.00 Hz, and 8.57 Hz, reaching an average accuracy between 97.22% and 100% and an average information transfer rate (ITR) between 15.42 bits/min and 33.92 bits/min. All participants successfully used the SSMVEP-based speller with all types of stimulation pattern. The most successful SSMVEP stimulus was the SSMVEP1 (pendulum-like movement), with the average results reaching 100% accuracy and 33.92 bits/min for the ITR.

## 1. Introduction

Human vision is the most important sense for us to recognize and understand the world around us; the perception of motion is the basic sensation that develops in the early stage of neural system development. A Brain–computer interface (BCI) is a system in which the user can, e.g., control or adjust an electronic device with neither the use of the peripheral nerves nor muscles; typically, utilizing an electroencephalography (EEG) recording. Within the variety of EEG-based BCI paradigms, the steady-state visual evoked potential (SSVEP) is one of the fundamental ones [1]; thanks to it’s straightforward implementation it is a common choice for speller oriented applications [2]. The typical SSVEP-based BCI uses a constant flickering light source, e.g., LEDs or objects displayed on a computer screen usually turning on/off with a stable frequency (typically 6–90 Hz) [3]. One of the fastest online SSVEP-based BCIs was presented by Chen et al. [4] in 2015. It reached an average accuracy and information transfer rate (ITR) of 99% and 267 bits/minute (bpm), with a peak ITR of 315 bpm. In the recent decade [5], achieving high accuracy with a high literacy rate (number of users for which the BCI achieved high accuracy) increased significantly [6]. In two studies with 61 participants, Gembler et al. [7] and Stawicki et al. [8] were able to achieve a 100% literacy rate, with an SSVEP-wizard selecting the optimal frequency for the BCI-speller, and robot steering, respectively. Despite the promising results of the SSVEP paradigm, one of the main downsides is fatigue, due to the constantly blinking stimulus [9]. This standard (SSVEP) approach can yet cause among others visual fatigue [10] and high mental load [11], as a result, creating an uncomfortable user experience. One of the solutions to prevent this unwilling response is, e.g., the use of motion-based stimulation, as suggested in 2016 in [11]. In order to reduce the unpleasant (side) effects of the standard SSVEP, some researchers proposed a constant motion-based approach for the stimuli.

The first research dedicated to investigate the motion evoked responses, dates back to 1952, where Marshall & Harden described their experiment with a pulsating circle stimuli, using a cathode ray tube—CRT display [12]. During the following decades, the motion based visual evoked potentials were broadly tested to determine the basics of motion perception. A brief summary with methodological description of the motion visual evoked potentials can be found in the 2007 review by Heinrich [13]. The recent development in the field of EEG-based BCI uses modern techniques to increase the user comfort of the visual evoked potentials (VEP) stimulation (e.g., SSVEP). Proposed research techniques involve a flicker-free stimulation to induce the desired brain responses (VEP). In recent years, there has been growing interest in steady state motion visual evoked potentials (SSMVEPs), especially based on the motion-reversal patterns. One of the proposed solutions for this specific (motion-reversal) behavior is the use of oscillating Newton’s rings [14,15,16], an oscillation (contraction-expansion) of a circled checkerboard pattern [17,18] or a swing motion, a radial rotation and a spiral rotation [19]. Xie et al. [14] tested six participants in an offline experiment with oscillating Newton’s rings-based stimulation. They achieved an (theoretical offline) accuracy of 86.6% and an ITR of 15.9 bits/min, utilizing the canonical correlation analysis (CCA) for target classification. In 2018, Guitong et al. [15] compared two different shapes of the stimulation objects, one version was a typical circled (concentric) Newton’s rings, the second one was squarish (mimicking the Newton’s rings projected on a square). The two versions of the stimuli were used in a concentric oscillating approach, in order to impersonate standard flickering frequency. Both versions utilize an 8-target BCI system with a CCA-based classification method. On average, eight participants achieved an accuracy of 85% and 85.9% for the squared and circled motion scenarios, respectively. For standard flickering frequencies, the participants reached an accuracy of 98.1% and 99.1% for the squared and circled stimulation forms, respectively. In 2017, Yan and co-authors examined an SSMVEP stimulation method, utilizing an equal-luminance red-green circular checkerboard pattern [17]. Nine subjects tested the stimulation pattern and a black-white checkerboard pattern, both with different frequencies and stimulant durations. In an offline analysis, authors compared the accuracies of the corresponding conditions between the two paradigms. For classification, the CCA and power spectrum density methods were used, reaching an average accuracy of 86.6% and 88.2%, respectively. In 2019, Yan et al. compared four SSMVEP stimulation patterns; a swing movement, a radial rotation, a six-thread spiral rotation, and a concentric circled checkerboard with radial contraction-expansion [19]. Ten participants tested the four motion scenarios with frequencies ranging from 7–12.8 Hz (linearly separated with an interval of 0.2 Hz). First, a set of offline data were collected to determine the optimal combination of template signal harmonics with the CCA classification method. In a follow-up online experiment, a 30-target BCI system (with trial and inter-trial times of 5 s and 1.5 s, respectively) utilizing the adopted CCA classifier was tested, reaching an average accuracy and ITR of 84.8% and 33.6 bpm for the swing movement, 80.9% and 31.9 bpm for the rotation motion, 95.3% and 41.2 bpm for the spiral scenario, and 80.7% and 32.6 bpm for the radical contraction-expansion checkerboard stimuli. In 2018, Han et al. tested a circled checkerboard pattern in an oscillating contraction-extraction motion in both offline and online experiments [18]. In the offline experiment, eighteen subjects had to look at the oscillating checkerboard pattern or a flickering stimuli with the motion inversion frequency or flickering frequency from 4.00–35.6 Hz, with 0.4 Hz increments. In the online BCI experiment, the participants gazed at the motion checkerboard scenario with 40 targets; each with a different motion inversion frequency, ranging from 7.0–14.8 Hz, in 0.2 Hz steps (utilizing the CCA method for frequency classification). For the online experiment, the trial duration varied between 2 and 4 s with an additional 0.5 s for gaze shifting. On average, all eighteen participants reached an accuracy of 94.0% and an ITR of 91.2 bpm, with a mean trial length of 2.8 s. In 2019, Chai et al. tested a radial zoom stimulation in a motion presentation pattern [20]. The authors compared this radial zoom stimuli to the typical on/off flickering and to the modulated Newton’s ring motion (e.g. [14]) integrated into an 8-target graphical user interface (GUI). The stimuli were divided into squared and circled forms. The flip frequencies of the 8 targets were between 8 and 15 Hz with Δf=1Hz. The recorded data were processed offline with the CCA utilized for target classification. In total, eight participants reached an average accuracy of 89.7% and 93.4% for the SSMVEP-square-zoom and the SSMVEP-circle-zoom versions, respectively. For the squared and circled Newton’s rings stimulations, the achieved accuracies were 76.3% and 77.5%, respectively. The highest offline ITR (42.5 bits/min) was reached for the circular radial motion stimuli with a classification time window of 3 s. In 2020, Zhang et al. presented an SSMVEP BCI approach utilizing a human gaiting sequence as a stimulus. In a four class BCI setup, the system achieved an average accuracy of 88.9% across ten subjects [21]. The authors showed that the gait stimulus generated an additional sensorimotor response. In our preliminary study in 2019, Stawicki et al. tested an SSMVEP based on vertical motion of a stimulus (pendulum behavior) [22]. Seven participants in a copy-spelling task with a 4-target system, achieved an average accuracies of 99.0%, 100%, and 98.7% utilizing the following frequency ranges: 3.0–3.75 Hz, 6.00–6.67 Hz, and 9.23–12.0 Hz, respectively. In another study, Volosyak et al. 2020 compared three major VEP stimulation approaches; the common frequency based SSVEP (also named fVEP), the m-sequence code based stimulus (cVEP), and the SSMVEP [6]. Besides the performance, the study tested also the personal preferences and demographic factors across 86 subjects, with a copy-spelling task. All 86 participants finished the cVEP with an average accuracy and ITR of 97.8% and 40.2 bpm, respectively. Eighty-three (83) subjects successfully accomplished the fVEP speller task with a mean ITR and accuracy of 31.9 bpm and 95.3%, respectively. The SSMVEP was successfully finished by 80 subjects with a mean ITR and accuracy of 26.4 bpm and 91.1%. Here, the SSMVEP stimulus was designed as a continuous vertically decreasing/increasing motion, creating an illusion of a flipping coin.

An overview of the recent studies implemented SSMVEP stimulations is presented in Table 1.

The SSMVEP can also be utilized for vision testing; in 2020, Zheng et al. compared six motion visual stimuli—oscilating expansion-contraction of concentric rings, reversal checkerboard, reverse vertical square-wave gratings, reversal horizontal and vertical sinusoidal gratings, and brief-onset vertical sinusoidal gratings—for objective visual acuity assestment [23]. The authors found differences in the harmonic component of the motion-based SSVEP response.

We realized some of the reported findings were particularly interesting, requiring further investigation. Our results of the CCA maximum coefficient spectrum differ from that of the literature (e.g., Han et al. 2018 [18]), we found out that some of the motion-based stimulation in fact do elicit a harmonic response, as it was always the case in all our previous studies. The presented study was designed and performed to explore and assess different SSMVEP1-5 motion behaviors (linear, radial, zooming), and their harmonic response in a practical usage scenario, through the comparison of the distinct/individual classifying accuracy and overall system speed (information transfer rate). In order to make a general assessment of the SSMVEP stimuli, including the new types SSMVEP4 and SSMVEP5 introduced in this paper, they all were directly compared with the typical SSVEP flickering stimuli. If the motion-based stimuli proves to be faster or more comfortable for users, they could influence visual stimulus designs in future research or application.

This paper is structured as follows: Methodology Section 2 followed by the result Section 3 and finally the discussion of our findings in the Section 4.

## 2. Materials and Methods

This section describes the hardware and software solutions of the reported study, all of which are necessary to replicate this experiment.

Ethics Statement

All participants (healthy young students) gave written informed consent. Information needed for the analysis was stored anonymously. This research was approved by the ethical committee of the medical faculty of the University Duisburg-Essen.

### 2.1. Subjects

The total number of participants were nine (3 males, 6 females, 0 diverse) with an average age of 24.7, ranging from 20–33 years, and a standard deviation (SD) of 4.30; all students of the Rhine-Waal University of Applied Sciences. The participants had normal or corrected-to-normal vision, 6 had previous experience with SSVEP-based BCI systems, and 8 were right-handed. The experiment took place in a regular laboratory room, while the light intensity was kept on an acceptable level (ambient indirect daylight). The experiment took approximately 60 min. All participants received a financial reward for their participation.

### 2.2. Questionnaire

The questionnaire for each tested stimulus contained eight questions, two of them were answered after a short familiarization run (spelling of the word “BCI”) and six were answered directly after spelling the word “INVITE” (which was the main task for further evaluations). In the questions, the participants had to subjectively assess their impression of the tested system on a Likert scale 1–7, where 1 means full agreement with one term and 7 the total disagreement (agreement with the opposite term). The terms to evaluate after the training spelling (“BCI”) were focused on the visual stimulation only: Relaxed—exhausting and comfortable—annoying. The question terms that followed the main spelling tasks addressed the user friendliness of the tested interface and the spelling system itself. The terms used for this were: Efficient—inefficient, clear—confusing, exciting—boring, inventive—conventional, enjoyable—annoying, and fast—slow. The motivation for these questions was the User Experience Questionnaire described in [24], similar to our recent study Volosyak et al. 2020 [6].

### 2.3. Visual Stimulation

The self-written custom-made BCI program (based on OpenGL and C++) was developed using Microsoft Visual Studio 2015 Community (Microsoft, Redmond, WA, USA) and presented the visual stimulation on a standard LCD monitor (24-inch, Acer Predator XB252Q) with a screen resolution: 1920×1080 pixels and the vertical refresh rate $V_{RR}$ of 240 Hz.

**Figure 1:**
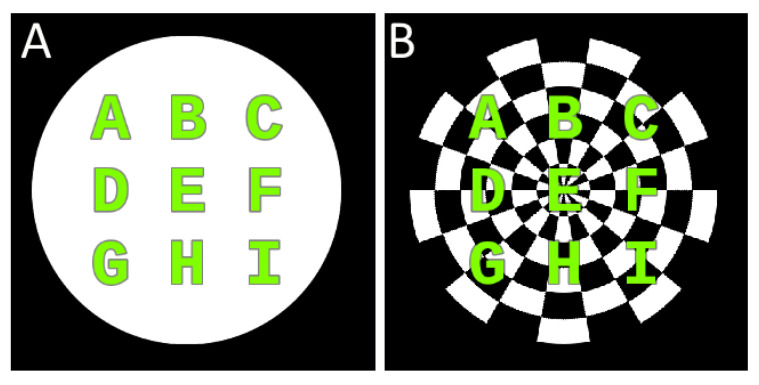
Stimulation circles used in the study, both were 270 pixels in diameter with the text content located inside the center. A white circle (**A**) and a circled checker-board consisting of 20 arcs and six layers (**B**).

There were two types of stimulation: Full color circle (SSVEP, SSMVEP1, SSMVEP2) and a checker-board circle (SSMVEP3–5), see Figure 1. Based on these stimulation objects, we investigated five movement patterns with the following motion designs:

- SSMVEP1-up-down movement oscillation,
- SSMVEP2-oscillating in vertical size (flipping illusion),
- SSMVEP3-checkerboard pulsation,
- SSMVEP4-arc’s inverse pulsation,
- SSMVEP5-arc’s inverse rotational oscillation.

The movement-speed of the tested stimulation patterns were generated utilizing the following base-frequencies: 7.06, 7.50, 8.00, and 8.57 Hz, and the trigonometric function $cos(\left(2\;\pi \;f_{K}\;i\right)/V_{RR})$, where $f_{K}$ is the base-frequency, *i* is the frame index, and $V_{RR}$ is the refresh rate of the monitor. The length/angle of the movement step/rotation were calculated depending on the number of frames in each cycle of the base-frequency of the corresponding movement behavior. The even number of frames (in a frequency cycle) ensured the same number of steps/angles in both direction (forward and reverse, clockwise and counterclockwise) of the motion (linear, radial, zooming)-behavior. The chosen frequencies shall meet the following conditions: have a smooth movement effect for better user experience, e.g., for the highest frequency 8.57 Hz the number of steps in one direction is 14 (with used $V_{RR}=240$ Hz), and for 3 Hz it would be 34 frames, and an induced, fairly strong harmonic response of the VEP stimulation (expect a relative strong harmonic response to certain stimuli designs) which is commonly known to be the case for lower stimulus frequencies [3].

SSVEP

**Figure 2:**
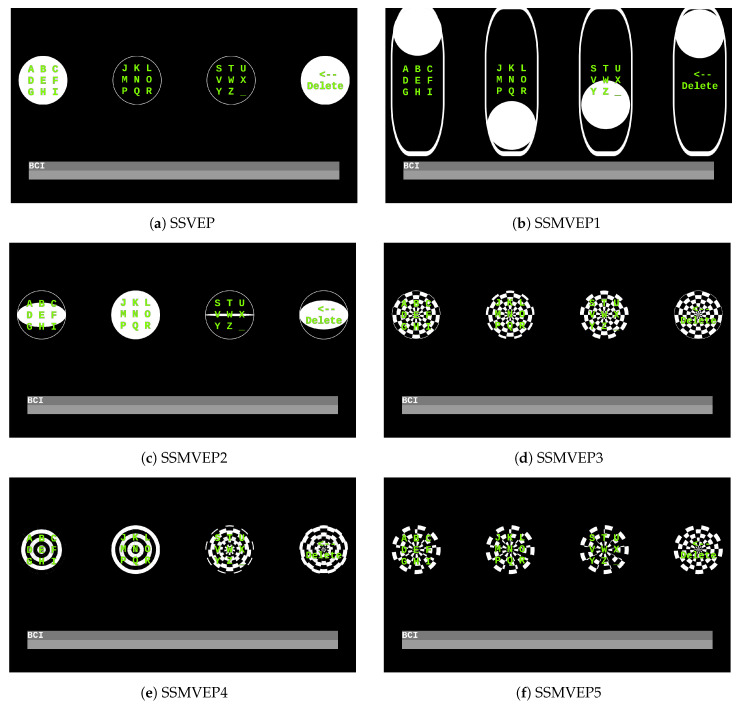
Different versions of flicker-free SSMVEP stimulus (working example) patterns evaluated in this study, presented during the stimulation in different phases. (**a**) Standard steady-state visual evoked potentials (SSVEP) stimulation during the stimulation. The middle boxes are in the off state and the outer boxes are in the on state. (**b**) The SSMVEP1 stimulation (pendulum behavior). The stimulation circle moves above and below it’s starting, central position. (**c**) The SSMVEP2 stimulation (flipping-coin behavior). The stimuli changes it’s vertical size during the stimulation. This motion pattern creates the illusion of a coin that rotates horizontally around its vertical axis. (**d**) The SSMVEP3 stimulation (pulsation behavior). The stimuli was composed of six layers of 20 degree black and circular sectors composed into a checkerboard. The motion consisted of contractions and expansions while keeping a constant ratio. (**e**) The SSMVEP4 stimulation (reverse (inverse) arcs pulsation behavior behavior). In this stimuli design each checkerboard group of arcs (20 degrees sector) moved in opposite directions (inwards and outwards) to its neighbor. (**f**) The SSMVEP5 stimulation (reverse (inverse) arcs rotation behavior). Here the 20 degree checkerboard arcs rotated 20 degrees in opposite direction.

In the SSVEP stimuli white colored full circle behind the characters was appearing and disappearing at a specific rate according to the utilized frequency. The stimuli were generated with the squared wave function based on $sin(2\;\pi \;f_{K}\;i/V_{RR})$, where $f_{K}$ is the frequency, *i* is the frame index, and $V_{RR}$ is the refresh rate of the monitor (see Figure 2a).

SSMVEP1

In the SSMVEP1 design, the position of the stimulation circle was based on the cosine function and the condition that the maximum and minimum cosine-based positions are above or below the original (center/starting) position, see Figure 2b.

SSMVEP2

In the SSMVEP2 design, the vertical scale (vertical size) of the stimulus changes stepwise, according to the base-function, see Figure 2c.

SSMVEP3

The design of the SSMVEP3 stimuli consisted of 6 layers of rotated black-white arcs (cutouts of a full circle). The contraction behavior in this design was applied to every layer in order to ensure equal contraction ratio of the whole stimuli, see Figure 2d.

SSMVEP4

The SSMVEP4 stimulus consisted of 6 layers of checkerboard arcs (cutouts of a full circle) stacked, the ratio of the displayed layers was kept constant during the opposite arcs oscillations, see Figure 2e.

SSMVEP5Ethics Statement

The SSMVEP5 was generated using the opposite rotation angle of the arcs-layer-based black-white circles, that were rotating stepwise in opposite directions, see Figure 2f.

### 2.4. Data Acquisition

The EEG was recorded with the g.USBamp (g.tec, Graz, Austria) USB-biosignal amplifier (sampling rate 600 Hz), connected to an Intel Core-i7-8700K @3.7GHz CPU based desktop PC running Microsoft Windows 10 Education (1809) (64-bit, Microsoft, Redmond, WA, USA) operating system. The used PC (Dell Precision 3630 Tower) was equipped with 16 GB RAM, and an NVIDIA GeForce GTX 1080 graphics card. Since the g.USBamp has 16 available signal inputs (besides ground and reference), passive EEG electrodes (g.LADYbirdPASSIVE, g.tec, Graz, Austria) were utilized, mounted on the positions available in the g.GAMMAcap${}^{2}$ (according to the international 10-10 system, extended with intermediate positions): $AF_{Z}$ (ground), $C_{Z}$ (reference), and the position of the 16 used signal electrodes were: $P_{Z}$, $P_{3}$, $P_{4}$, $P_{5}$, $P_{6}$, $PO_{3}$, $PO_{4}$, $PO_{7}$, $PO_{8}$, $O_{Z}$, $O_{1}$, $O_{2}$, $O_{9}$, $O_{10}$, $POO_{1}$, and $POO_{2}$.

Abrasive electrode gel was used to bring the impedances below 5 kΩ. A digital notch filter around 50 Hz was applied before the data were further processed.

### 2.5. Data Analysis

An overwhelming majority of the reported studies utilizes the CCA method for data analysis (e.g., [14,15,18,19,20,21]). Based on our previous experience with the minimum energy combination (MEC) method [7,8,25,26], we decided to evaluate it with the filter banks (FB) in a practical scenario (spelling task), similar to our previous studies implementing the SSMVEP1 or SSMVEP2 stimuli, where the FB-CCA [22] or CCA [6] methods were utilized.

#### 2.5.1. Minimum Energy Combination (MEC)

The MEC uses principal component analysis (PCA) to cancel out components that do not contribute to the stimulus response.

The response to a specific stimulus frequency *f*, as well as its $N_{h}$ harmonics can be defined as the voltage between the *i*-th electrode and a reference at time *t*, which is subjected to a phase-shift and channel specific environmental nuisance and noise signal $E_{i}$,

(1) $$y_{i}\left(t\right)\;=\;\sum_{k=1}^{N_{h}}a_{i,k}\sin\left(2\pi kft\right)+b_{i,k}\cos\left(2\pi kft\right)+E_{i}\left(t\right).$$

Generally, we consider *M* samples of EEG data, recorded for each of *N* signal electrodes at a sampling frequency of $F_{s}$ Hz. Let $N_{h}$ denote the number of harmonics that are considered for classification; for each stimulus frequency $f_{i}$, i=1,…,K a reference matrix $R_{f_{i}}\in R^{2N_{h}\times M}$ is constructed as

(2) $$R_{f_{i}}=\left[\begin{array}{c} \sin(2\pi f_{i}t)\\ \cos(2\pi f_{i}t)\\ \vdots \\ \sin(2\pi N_{h}f_{i}t)\\ \cos(2\pi N_{h}f_{i}t) \end{array}\right],\;t=\frac{1}{F_{s}},\frac{2}{F_{s}},\ldots ,\frac{M}{F_{s}}.$$

For the reference $Y\in R_{f_{1}},\ldots ,R_{f_{K}}$ corresponding to the fixed stimulation frequency *f* and the EEG signal matrix $X\in R^{N\times M}$ holding the data for classification, we can generalize (1) to $X^{T}=Y^{T}A+E$, where the phases and amplitudes corresponding to *Y* are stored in the matrix $A\in R^{2N_{h}\times N}$ and the noise signals are stored in $E\in R^{M\times N}$.

The goal of the MEC is to amplify the SSVEP-amplitudes and filter out the noise signal.

For this, the noise matrix *E* is estimated using orthogonal projection $\tilde{E}=X-Y{\left(Y^{T}Y\right)}^{-1}Y^{T}X$. Now, a vector $\hat{w}$ that minimizes the energy of $\tilde{E}$ is searched,

(3) $$\min_{\hat{w}}{\|\tilde{E}\hat{w}\|}^{2}=\min_{\hat{w}}{\hat{w}}^{T}{\tilde{E}}^{T}\tilde{E}\hat{w}.$$

This optimization problem can be solved by calculating the eigenvalues $\lambda_{1}\leq \lambda_{2}\leq \ldots \lambda_{N}$ and the corresponding eigenvectors $v_{1},\ldots ,v_{N}$ of ${\tilde{E}}^{T}\tilde{E}$, which are then used to define a set of weight vectors,

(4) $$w_{i}=\frac{v_{i}}{\sqrt{\lambda_{i}}},\;i=1,\ldots ,N,$$

and further, yielding a set of virtual channels (components)

$$s_{i}=X^{T}w_{i},\;i=1,\ldots ,N.$$

To detect the SSVEP response for the specific frequency *f*, the power of that frequency and its harmonics $N_{h}$ is estimated by

(5) $$\hat{P}\;=\;\frac{1}{N_{s}N_{h}}\sum_{l=1}^{N_{s}}\sum_{k=1}^{N_{h}}{\|X_{k}s_{l}\|}^{2},$$

where $X_{k}\in R^{2\times N}$ is defined as the sub-matrix of *X* constructed from the rows containing the sine and cosine data of the *k*-th harmonic [26]. These SSVEP power estimations are computed for all *K* considered frequencies yielding the probabilities

$$p_{i}\;=\;\frac{\hat{P_{i}}}{\sum_{j=1}^{N_{f}}{\hat{P}}_{j}},\;i=1,\ldots ,K.$$

#### 2.5.2. Filter Banks (FBs)

As a last step, a filter bank method utilizing an 8th order Butterworth filter was applied (see example in [27]). The lower and upper cut-off frequencies of the *m*-th sub-band were: $FB_{1}$ 5.00–90.00 Hz, $FB_{2}$ 12.00–90.00 Hz, $FB_{3}$ 19.00–90.00 Hz, $FB_{4}$ 26.00–90.00 Hz, and $FB_{5}$ 33.00–90.00 Hz. Here, five sub-bands were analyzed, i.e., the MEC was used for each individual sub-band component, yielding a set of probabilities $p_{k}^{1},\ldots ,p_{k}^{5}$, k=1,…,K. The target identification label *C* was determined as linear combinations of these sub-band probabilities,

(6) C=arg maxk=1,…,Kp˜k,where p˜k=∑m=15ampkm.

The weights $a_{m}$ in Eq. (6) were set to: $a_{1}=0.4$, $a_{2}=0.2$, $a_{3}=0.15$, $a_{4}=0.13$, and $a_{5}=0.11$, as proposed in [27]. A value of ΔC was defined as the distance between the highest and second highest ${\tilde{p}}_{k}$, for k=1,…,K. The BCI output corresponding to *C* was only produced if $\Delta C>\beta_{T}$. This distance-based classification criteria was successfully tested with the CCA [6] and FB-CCA [22] method. Moreover, in this experiment, the number of considered harmonics was set to $N_{h}=5$, the number of signal channels was N=16 (see Signal acquisition) and the number of stimuli classes was K=4.

### 2.6. Classification Window

The data were classified online every 0.25 s, with each new data block, for the time window higher or equal to the minimal time window (1 s). The sliding window technique was introduced in order to collect up to 64 blocks of data (maximum time window of 16 s), with the classification attempts calculated every block on the collected data. After reaching the maximum 64 blocks of data, the oldest EEG data block was shuffled out, the data were shifted and a new block was added at the end of the 16 s time window. This block length of 0.25 s and the maximum time window of 16 s were chosen based on preliminary tests, the amplifier data transfer rate, and the computational load of the FB-based MEC classification technique.

This approach was based on our previous studies with the sliding window technique [7,8], under the assumption, that the visual response will build up (till some level) as long as the user continues to gaze at the stimulus.

### 2.7. Procedure

Participants were seated in a comfortable chair and instructed to stare (fix their gaze) at the center of the target (circle or circled checkerboard pattern) containing the desired letter and not to follow the movements of the SSMVEP stimuli. Once the details of the experiment were explained, the consent form was signed, the participants were prepared for the EEG recording experiment. The starting value of the personal-dependent threshold ($\beta_{T}=0.3$) was empirically determined with a number of pre-tests in order to achieve the best accuracy and typing speed. Afterwards, the threshold ($\beta_{T}$) was manually adjusted (if needed) during a short introduction (few selections) and confirmed by a repeated selection of each target two times. In a total of 9 cases (sessions) the $\beta_{T}$ was lowered to 0.29 for both spelling tasks. The order, in which the different SSMVEP patterns were tested (sessions), was randomized, as well as the frequency arrangement of the stimuli (shuffled before each spelling task). First, the participants were asked to type a short word “BCI” (9 trials) with a fixed selection time parameters (the minimum and maximum time windows for a selection were set to 4 s; 16 blocks of data) and an additional red arrow was pointing at the proper target. Since the letters were visible the whole time the user could learn the interface behavior. After this short training round, the participants were asked to answer the two questions about the visual stimulation. Afterwards, the main spelling task $T_{S}$ was started. Here, the user was asked to write the word “INVITE” (18 trials) without the red arrow guiding the user as she/he needed to select the proper letter or group of letters by looking at the stimuli behind the letters. For this main task, the classification time window varied between 1 and 16 s (see Section 2.6). After finishing the main task, the participants were asked to answer the six questions regarding the tested system. The main task was used for the evaluation of the system performance. In total, 2 spelling tasks (session) were carried out by every participant. After every target classification (performed selection), a short break was introduced, to allow users to shift their gaze to the next target (gaze shift or pause). This pause time was set to 2 s in both tasks. The time between the different SSMVEP patterns varied between the participants, while some participants were eager to test the next SSMVEP stimuli design (next session) after just 30 s, others waited up to 8 min between the sessions. In the main task (spelling “INVITE”), the frequencies $f_{1}$, $f_{2}$, and $f_{3}$ were selected approximately the same number of times, if no errors were made. For more details regarding the spelling behavior of the GUI see [6].

### 2.8. Information Transfer Rate (ITR)

The overall performance of the tested scenarios was calculated using the popular information transfer rate (ITR) formula, introduced in [1]:

(7) $$B_{T}=\frac{60}{T}\times C_{N}\times \log_{2}N+P\log_{2}P+\left(1-P\right)\log_{2}\left[\frac{1-P}{N-1}\right],$$

(8) $$B_{M}=\frac{60}{T}\times C_{N}\times B_{T},$$

$B_{T}$ is the ITR in bits per trial, *P* is the accuracy of the experiment, *T* is the total time of the experiment, $C_{N}$ is the total number of target selections in the experiment, *N* is the number of targets (here, N=4), and $B_{M}$ represents the ITR in bits per minute. An online ITR calculator can be found at (https://bci-lab.hochschule-rhein-waal.de/en/itr.html). The maximum possible ITR that can be reached with this setup is 40.00 bits/min (min. classification window of 1 s + 2 s for gaze shift).

### 2.9. Statistical Analysis

In order to test the statistical significance, we utilized a one-factor ANOVA with repeated measurements. In some cases, the non-parametric equivalent of the ANOVA was used (Friedman’s test). Additionally, a paired *t*-test was applied to determine which pairwise groups were significantly different. The significance level was *p* < 0.05 for all tests.

## 3. Results

In this paper we present the concept of a new design for the flicker-free steady-state motion visual evoked potentials, evaluate them with well established methods, and compare them to previously reported findings. The performance results of the online experiment, the accuracies and the information transfer rates (ITRs) Equation (8), are presented in the Table 2. In this study we extend the previous findings, SSMVEP1 vs. SSVEP in [22] and SSMVEP2 vs. SSVEP vs. cVEP in [6], in a comprehensive comparison across them and the novel stimulus (SSMVEP4 and SSMVEP5) designs.

All participants were able to successfully finish the spelling tasks hence the literacy/efficiency rate was 9/9 for all tested stimulation scenarios. The most successful SSMVEP stimulus was the SSMVEP1 (pendulum-like movement), with the average results reaching 100% accuracy and 33.92 bits/min for the ITR.

The average (SD) times of the experimental task $T_{S}$ (spelling of the word “INVITE” in 18 trials) were: 64.1 s (9.2) for the SSVEP, 63.4 s (12.2), 80.8 s (21.9), 123.2 s (36.5), 152.5 s (57.5), and 116.8 s (41.7), for the SSMVEP1 to SSMVEP5, respectively.

**Figure 3:**
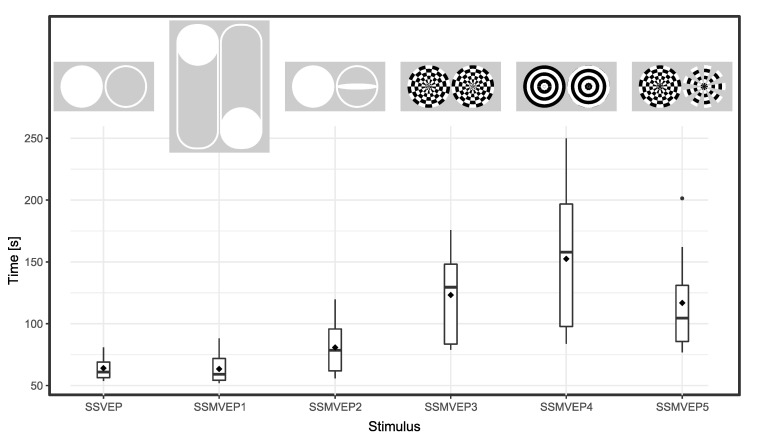
Spelling times of the word “INVITE” which was the main task consisting of 18 trials. The bar inside the box represents the median value and the ⋄ points the mean values. Above the results presented are the stimuli at the beginning and in mid-cycle of the stimulation period.

A detailed Box-Graph presenting the total times of the “INVITE” task is shown in Figure 3.

Statistical Tests

In order to verify the statistical analysis, a one factor repeated measurement ANOVA was used. Since the accuracy results violate the normality condition of the ANOVA, the Friedman’s test (non-parametric equivalent of the ANOVA) was used to compare the accuracy, resulting in p=0.177. Since no accuracy differences were found, we can not reject the null $H_{0}$.

In order to check the ITR and total time results for statistical significance, a detailed pairwise comparison (*t*-test) was applied and the resulting p-values are reported in Table 3.

Questionnaire Results

We tested the statistical significance of the questionnaire results separately for each question, with Friedman’s test. The results showed that the users responses to the “efficient”, “exciting”, “enjoyable” and “fast” questions (options) differed significantly between the tested stimuli, with the *p*-values p<0.001, p<0.001, p<0.05, and p<0.01, respectively. In order to further analyze the results, we used pairwise comparison-using Wilcoxon signed-ranks test (WSR), see Supplementary Figure S3. The user ratings, in the case of visual stimulation, mostly did not differ significantly, yet the individual ratings regarding the system did (see Figure S3). Based on the user ratings, the users did not find the systems to be more exhausting. Most of the participants rated the SSMVEP systems as the clear ones, especially the SSMVEP1, which received same ratings as the SSVEP scenario, with an average scores of 1.1 and 1.2 for SSVEP and SSMVEP1, respectively). Most of the SSMVEP systems were also rated as the more inventive ones, SSVEP score 1.8 and SSMVEP average scores of 1.7, 1.6, 2.1, 2.2, 2.2 for SSMVEP1–5, respectively. Almost all tested systems were subjectively rated as fast ones besides SSMVEP4 (checkerboards radial contraction-expansion motion), which received an almost moderate score of 3.4 between fast and slow. SSVEP score was 1.1, SSMVEP1–3 reached 1.6, 1.6, 2.0, respectively, and SSMVEP5 was scored 2.9. These ratings showed significant differences between SSVEP, SSMVEP1, SSMVEP2, and SSMVEP3 against SSMVEP4 with p<0.05 (WSR). Moreover the differences in ratings between SSMVEP3 and SSMVEP5 were significant (WSR p<0.05). In the case of exciting vs. boring ratings, the participants rated on average (scores in brackets), the SSMVEP1 (1.9) and SSMVEP2 (1.8), together with the SSVEP (2.1), as more exciting than the SSMVEP3 (2.9), SSMVEP4 (3.7), and SSMVEP5 (3.2). A pairwise signed-ranks test, showed significant differences in favor of SSVEP, SSMVEP1, and SSMVEP2 against SSMVEP 4 and SSMVEP5 (WSR p<0.05). While ranking the enjoyable vs. annoying question, the users rated the SSMVEP4 system as the least enjoyable (3.8) amongst all tested versions with scores of 2.2 for SSVEP, 2.2 for SSMVEP1, 2.2 for SSMVEP2, 3.1 for SSMVEP3, and 3.0 for SSMVEP5. These ratings were significant for SSMVEP4 against the SSVEP and SSMVEP4 vs. SSMVEP2 (WSR p<0.05). All other SSMVEP systems, including the SSVEP were found to be fairly enjoyable by the users. In the case of subjective response to efficiency, the highest scores, besides the SSVEP (1.3), were received by the SSMVEP1 (1.4) and SSMVEP2 (1.2) systems. Here, a statistical significance was found between the SSMVEP1 vs. SSMVEP5 (WSR p<0.05), and SSVEP vs. SSMVEP5 (WSR p<0.05). The other SSMVEP systems were rated as fairly efficient, with the average scores of SSMVEP4 2.6, and SSMVEP5 2.3.

## 4. Discussion

We tested five different SSMVEP stimulations with different designs and behaviors, two designs were moving in the vertical axis (one with an up and down movement, while the other one changed its vertical size), one design represented radial oscillation movements, and two represented radial movements of an arc’s segment (one rotational movement and one oscillation movement). All were compared to the standard SSVEP on–off appearance. In order to test a practical implementation of this novel stimulus, all designs were utilized in a four target 3-step spelling application and the users were asked to type a specific word with this BCI system.

We compared the five different SSMVEP stimuli to the traditionally SSVEP flickering and evaluated the accuracy, performance and questionnaire-based user friendliness for each of them. Some of the tested designs were demonstrated in our previous publications (SSMVEP1 in [22], SSMVEP2 in [6]) or literature SSMVEP3 in, e.g., [11,17,28], the SSMVEP4 and SSMVEP5 are novel designs.

Our results show that one of the presented SSMVEP scenarios (SSMVEP1) is as fast and accurate as the standard SSVEP. This scenario consisted of a translational motion (up & down). These findings confirm our preliminary study results presented in [22]. The second best performance results were achieved with the SSMVEP2 design. The accuracies did not differ significantly between any of the tested systems, all users were able to achieve close to perfect accuracies (>97%). The statistical results of the achieved accuracies show that all tested SSMVEP stimuli were as accurate as the standard SSVEP stimulation. This proves that all of the tested SSMVEP stimuli can easily induce a strong steady-state response [29], as discussed in [18,19,20]. We also examined a broader frequency response of the tested SSMVEP designs, where we compared the responses with an additional recording utilizing the CCA maximum correlation coefficient spectrum (see Figure S4). This spectrum shows, that the tested stimuli (SSMVEP1, SSMVEP2, SSMVEP3, and SSMVEP4) can also induce a high harmonic response, which is contrary to the findings of Han et al. [18], where a similar stimulation design to SSMVEP4 was examined.

Our findings on the subjective discomfort, that the SSMVEP approach is less exhausting and more comfortable—according to the questionnaire—are in-line with the literature [20]. We did not find any significant differences between the user questionnaire responses of the SSVEP against SSMVEP1 or SSMVEP2 (details in Supplementary Figure S3 and Table S1). This means, the motion behavior with similar performance to SSVEP (SSMVEP1) is not subjectively better than the SSVEP. On the other hand, even though the SSMVEP2 was not faster than SSVEP, it received a subjectively comforting user rating on average. The importance of this can be extended, the SSMVEP1 was as good as the SSVEP, thus it could replace the flickering approach in applications that have sufficient space on the screen for the movement representation, or even better, by utilizing the SSMVEP2 instead of the SSVEP, where the user comfort and preferences are traded off against the performance of the system.

Interestingly, while the translational movement based stimulations (SSMVEP1, SSMVEP2, SSMVEP3, and SSMVEP4) the harmonic component is fairly visible, in the rotational based stimuli (SSMVEP5) only the fundamental component is visible in the CCA maximum coefficient spectrum (see Figure S4). This observation confirms the findings of [19], where the rotation based SSMVEP did not induce any visualizable harmonic response in the presented spectrum.

When comparing the checkerboard stimuli presented in this study with the ones tested in recent literature [18,19], we can see that the designs tested in this study consisted of smaller number of stimuli and slightly reduced number of single elements in the checkerboard pattern. In the GUI design, the letter labels partially covered the stimulation surface; this could have had an impact especially on the circulated checkerboard designs (SSMVEP3, SSMVEP4, and SSMVEP5). Only frequencies between 6 and 8 Hz were examined, hence, other ranges could be explored, particularly frequencies from 4 to 6 Hz. The tested frequency range (7.06–8.57 Hz) was chosen based on our preliminary study [22] as a trade-off between speed and accuracy. The lower performance and thus the long spelling time of the SSMVEP4 stimuli might influence the subjective user comfort response against this design.

Further research should compare the FB-CCA and FB-MEC methods and investigate the influence of the FB composition on the SSMVEP classification, e.g., how many FBs are sufficient and which weight combination is optimal. Our results show that for future applications, the SSMVEP1 and SSMVEP2 should be focused on, as they induced the highest performance and best user rating results. A combination of the SSMVEP1 & SSMVEP2 could also be further investigated, with the possibility of providing a great user experience/comfort as well as a satisfactory performance. Most of the compared SSMVEP designs (SSMVEP1, SSMVEP2, SSMVEP3, and SSMVEP4) were implemented based on translational movement, however, the SSMVEP5 on the other hand, was based on rotational movement. This rotation-based stimulus requires further investigation with different designs and additional examination of the harmonic components. Future research in this area should also consider implementing the duty cycle, in order to increase the user’s comfort, by changing the movement ratio in a cycle, similar to SSVEP duty cycle, where a stimuli is not 50% on and 50% off but, e.g., 20% on and 80% off in a cycle.

The findings of this study suggest that SSMVEP can be applied when SSVEP is found to be annoying, confusing or destructive [20] like in a virtual reality environment, while keeping the overall performance at a similar level to the SSVEP. For this application, our results suggest that the optimal movement would incorporate translational components in their movement behaviors. For example, in VR applications, a motion-based stimuli could blend into the VR environment and still be easily detectable by the BCI system.

## 5. Conclusions

We tested the motion evoked potentials to find a more comfortable alternative to flickering visual stimulation. While we found one motion-based scenario (SSMVEP1) that was equivalent to the SSVEP regarding performance, none of the tested stimuli was found to be subjectively more comfortable than the traditional flickering stimuli. Our results confirmed previous findings, that oscillatory motion induces a steady-state response and have extended the research field of the flicker-free steady-state motion-based visual evoked potentials for further stimulus designs and evaluation. Our results suggest that future experiments implementing different translational and/or “flipping” motion-based behaviors, have a high chance of finding a more comfortable alternative. Based on the achieved accuracy and ITR performance results, supported by the positive user comfort ratings, we recommend replacing the uncomfortable on–off flickering with the motion-based stimuli approach.

## Figures and Tables

**Table 1:** Recent studies implementing motion-based visual evoked potentials (VEP) stimuli.

First Author	Year	Stimulus Design (Shape)	Behavior	Frequencies	Method
Xie [14]	2012	Newton’s rings (◯)	Reversal motion /translation	8.1, 9.8, 12.25, 14 Hz	CCA (offline)
Guitong [15]	2018	Newton’s rings Solid (◯/☐)	Reversal motion /translation	8–15 Hz (Δ *f* 1 Hz)	CCA (offline)
Yan [19]	2018	Checkerboard (◯) Spiral (◯) Swing (∇) Osscilation (◯)	translational rotation	7–12.8 Hz (Δ *f* 0.2 Hz)	CCA (online)
Han [18]	2018	Checkerboard (◯)	Reversal /translation	7–14.8 Hz (Δ *f* 0.2 Hz)	CCA (online)
Chai [20]	2019	Newton’s rings Solid box (☐)	Zoom motion translation	8–15 Hz (Δ *f* 1 Hz)	CCA (offline)
Zhang [21]	2020	Solid Circle (◯) Checkerboard (◯) Gating (Video frame)	Translational	8.57, 10 12, 15 Hz	CCA (online)
Stawicki [22]	2019	Solid circle (◯)	Translational up-down	3.0–3.75 Hz 6.00–6.67 Hz 9.23–12.0 Hz	CCA (online)
Volosyak [6]	2020	Solid circle (◯)	Translational Vertical zoom	8, 10, 12, 15 Hz	CCA (online)

**Table 2:** Results of the online SSMVEP experiment (spelling of the word “INVITE” in 18 trials); presented below are the average values of the accuracy (Acc) and the information transfer rate (ITR) of each individual subject, for all tested SSMVEP stimulation methods.

Subject	SSVEP		SSMVEP1		SSMVEP2		SSMVEP3		SSMVEP4		SSMVEP5	
	Acc [%]	ITR [bpm]	Acc [%]	ITR [bpm]	Acc [%]	ITR [bpm]	Acc [%]	ITR [bpm]	Acc [%]	ITR [bpm]	Acc [%]	ITR [bpm]
1	100	30.42	100	23.97	100	22.10	100	19.64	100	10.87	100	24.65
2	100	34.84	100	34.98	100	26.83	100	25.26	90.9	18.84	100	19.20
3	100	30.53	100	29.24	95.0	19.11	87.5	12.07	90.9	9.09	100	13.17
4	100	29.04	100	35.34	100	23.74	100	15.40	100	12.67	88.5	9.96
5	100	38.31	100	40.00	100	37.40	100	25.34	100	25.19	100	26.34
6	100	37.00	100	38.40	100	33.82	87.5	10.94	100	18.23	100	16.24
7	100	34.35	100	28.33	100	31.42	100	16.43	100	13.51	100	20.28
8	100	38.74	100	39.63	100	35.27	100	26.67	100	21.65	100	27.43
9	100	26.06	100	35.41	100	17.76	100	12.16	95.5	8.71	100	21.31
Mean	100	33.26	100	33.92	99.44	27.49	97.22	18.21	97.47	15.42	98.72	19.84
SD	0	4.45	0	5.56	1.67	7.27	5.51	6.25	4.01	5.82	3.85	5.92

**Table 3:** Statistical results, *p*-values and mean differences, of the ITR performance measurement of the main task (18-trials) using paired *t*-test.

Group 1	Group 2	ITR		Time	
		*p*-Value	Mean	*p*-Value	Mean
SSVEP	SSMVEP1	p=0.706	00.665	p=0.885	00.613
SSVEP	SSMVEP2	p<0.001	05.764	p<0.01	16.751
SSVEP	SSMVEP3	p<0.001	15.046	p<0.001	59.172
SSVEP	SSMVEP4	p<0.001	17.840	p<0.001	88.401
SSVEP	SSMVEP5	p<0.001	13.414	p<0.01	52.793
SSMVEP1	SSMVEP2	p<0.05	06.428	p<0.05	17.364
SSMVEP1	SSMVEP3	p<0.01	15.710	p<0.01	59.785
SSMVEP1	SSMVEP4	p<0.001	18.504	p<0.001	89.014
SSMVEP1	SSMVEP5	p<0.001	14.079	p<0.01	53.407
SSMVEP2	SSMVEP3	p<0.01	09.282	p<0.01	42.421
SSMVEP2	SSMVEP4	p<0.001	12.076	p<0.001	71.650
SSMVEP2	SSMVEP5	p<0.05	07.651	p<0.05	36.043
SSMVEP3	SSMVEP4	p=0.10	02.794	p=0.063	29.230
SSMVEP3	SSMVEP5	p=0.35	01.631	p=0.642	06.378
SSMVEP4	SSMVEP5	p=0.055	04.425	p=0.119	35.608

## Supplementary Materials

The following are available online at https://www.mdpi.com/2076-3425/10/10/686/s1, Figure S1: Overview of the SSMVEP1 and SSMVEP2 stimulus concepts., Figure S2: Overview of the SSMVEP3, SSMVEP4, and SSMVEP5 stimulus concepts., Figure S3: Participants’ subjective questionnaire responses., Figure S4: CCA maximum correlation coefficient spectrum., Table S1: Average results of participants’ subjective questionnaire responses, on a scele 1–7.

## Abbreviations

The following abbreviations are used in this manuscript:

EEG	electroencephalography
SSVEP	steady-state visual evoked potentials
SSMVEP	steady-state motion visual evoked potentials
CCA	canonical correlation analysis
MEC	minimum energy combination
bpm	bits/min
