# Supplementary material for: Comparison of Modern Highly Interactive Flicker-Free Steady State Motion Visual Evoked Potentials for Practical Brain–Computer Interfaces
Source: Brain Sci. 2020 Sep 28;10(10):686. doi: 10.3390/brainsci10100686 (PMC7601073; doi:10.3390/brainsci10100686)
Supplement: Supplementary file 1 [file brainsci-10-00686-s001.pdf]

brain sciences

MDPI

Comparison of Modern Highly Interactive Flicker-Free Steady State Motion Visual Evoked Potentials for Practical Brain-Computer Interfaces

Supplementary Figures

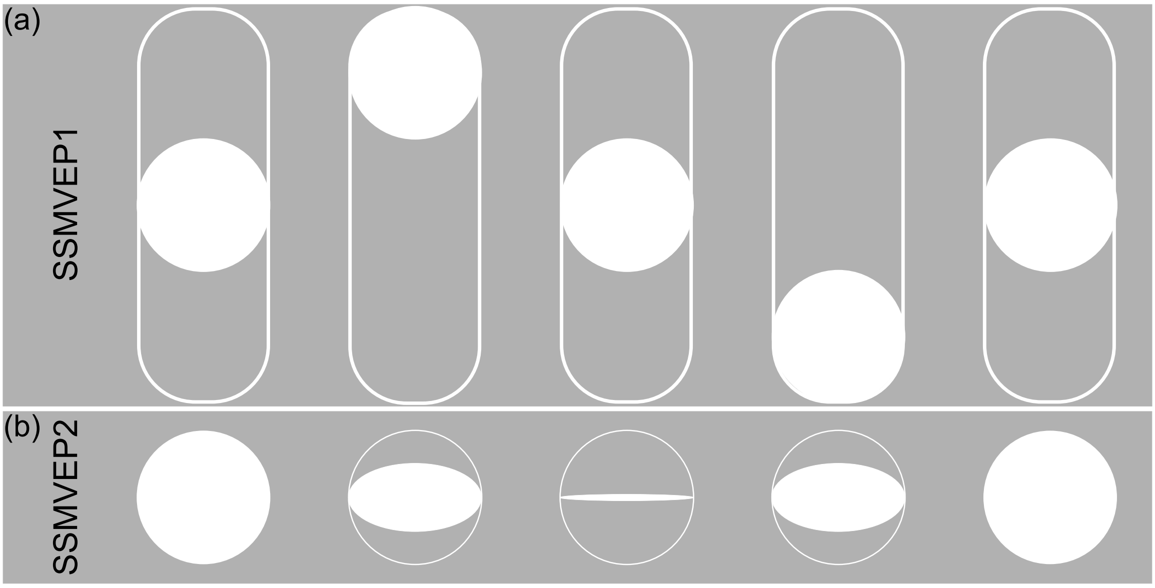

Figure S1: Overview of the SSMVEP1 and SSMVEP2 stimulus concepts used in the study. **(a)** Pattern of the SSMVEP1 stimulus. The stimulation circle moves in the vertical direction (up and down) above and below it's starting (center) position, shown on the left stepwise for one frequency period. Depending on the stimulation frequency, the length of the movement step varied. **(b)** Pattern of the SSMVEP2 stimulus presentation. Presented is a full period of the stimulation behavior. On the tested 240 Hz monitor this figure represents the 1st, 9th, 17th and 25th frames of a 7.5 Hz motion cycle with the last column starting with the 1st frame of a new cycle (full cycle consists of 32 frames).

Table S1: Average results of participants' subjective questionnaire responses, on a scele 1–7.

1	–	7	SSVEP	SSMVEP1	SSMVEP2	SSMVEP3	SSMVEP4	SSMVEP5
Relaxed	vs	Exhausting	2.33	3.33	2.44	3.44	3.00	3.00
Comfortable	vs	Annoying	2.44	3.22	2.56	3.78	3.22	3.33
Efficient	vs	Innefficient	1.33	1.44	1.22	1.89	2.56	2.33
Enjoyable	vs	Annoying	2.22	2.44	2.22	3.11	3.78	3.00
Exciting	vs	Boring	2.11	1.89	1.78	2.89	3.67	3.22
Fast	vs	Slow	1.67	1.56	1.56	2.00	3.44	2.89
Inventive	vs	Conventional	1.78	1.67	1.56	2.11	2.22	2.22
Clear	vs	Confiusing	1.11	1.22	1.33	1.44	1.67	2.00

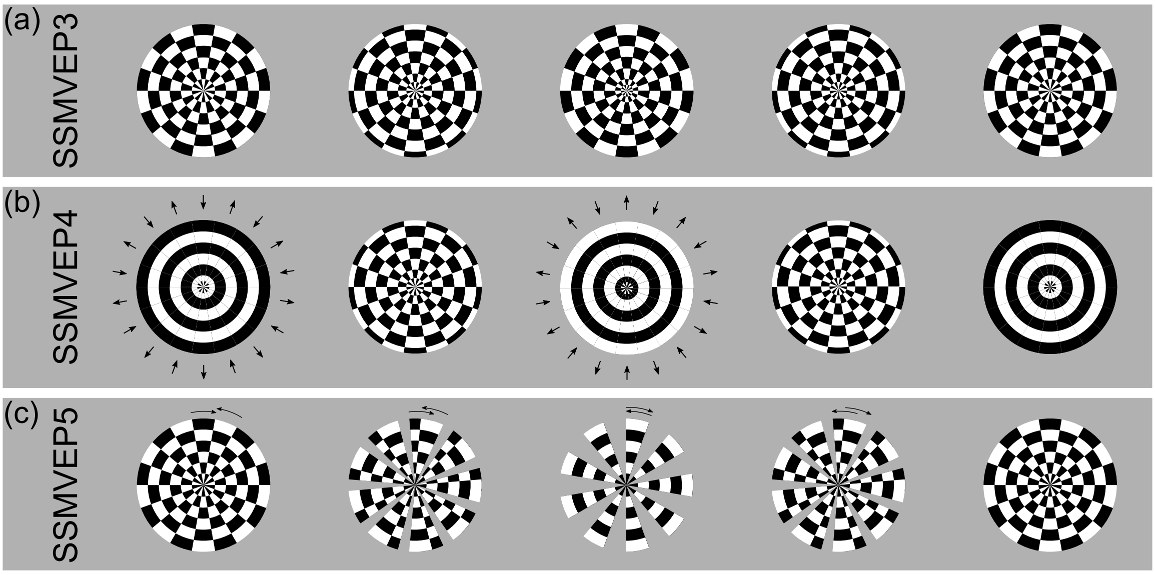

Figure S2: Overview of the SSMVEP3, SSMVEP4, and SSMVEP5 stimulus concepts used in the study. **(a)** Pattern of the SSMVEP3 stimulus presentation. This checkerboard stimulation pattern increases and decreases its inner sizes in a pulsation pattern, keeping the ratio between the layers constant. The checkerboard was created from a 20 degree circular sectors (arcs), each divided into six layers. A background layer of the stimulation pattern is visible during the pulsation, completing the outer ring of the checkerboard pattern. The center of the checkerboard is stable and the most inner layer is squeezed into it. **(b)** Pattern of the SSMVEP4 stimulus presentation. Neighboring circular sectors $\triangledown$ (arcs), each 20 degrees and divided into six layers, were moving in opposite directions (inwards and outwards). This checkerboard arcs patterns were pulsating in opposite directions in the rhythm of the stimulation frequency, creating an illusion of interchanging black/white rings. During the pulsating stimulation, the arcs kept the ratio between the rings constant and a checkerboard background pattern (completing the outer ring during the motion) was visible. **(c)** Pattern of the SSMVEP5 stimulus presentation. These checkerboard arcs (each 20 degrees and divided into six layers) patterns were rotating (20 degrees) in opposite directions in the rhythm of the stimulation frequency. On the tested 240 Hz monitor this figure represents the 1st, 9th, 17th and 25th frames of a 7.5 Hz motion cycle with the last column starting with the 1st frame of a new cycle (full cycle consists of 32 frames).

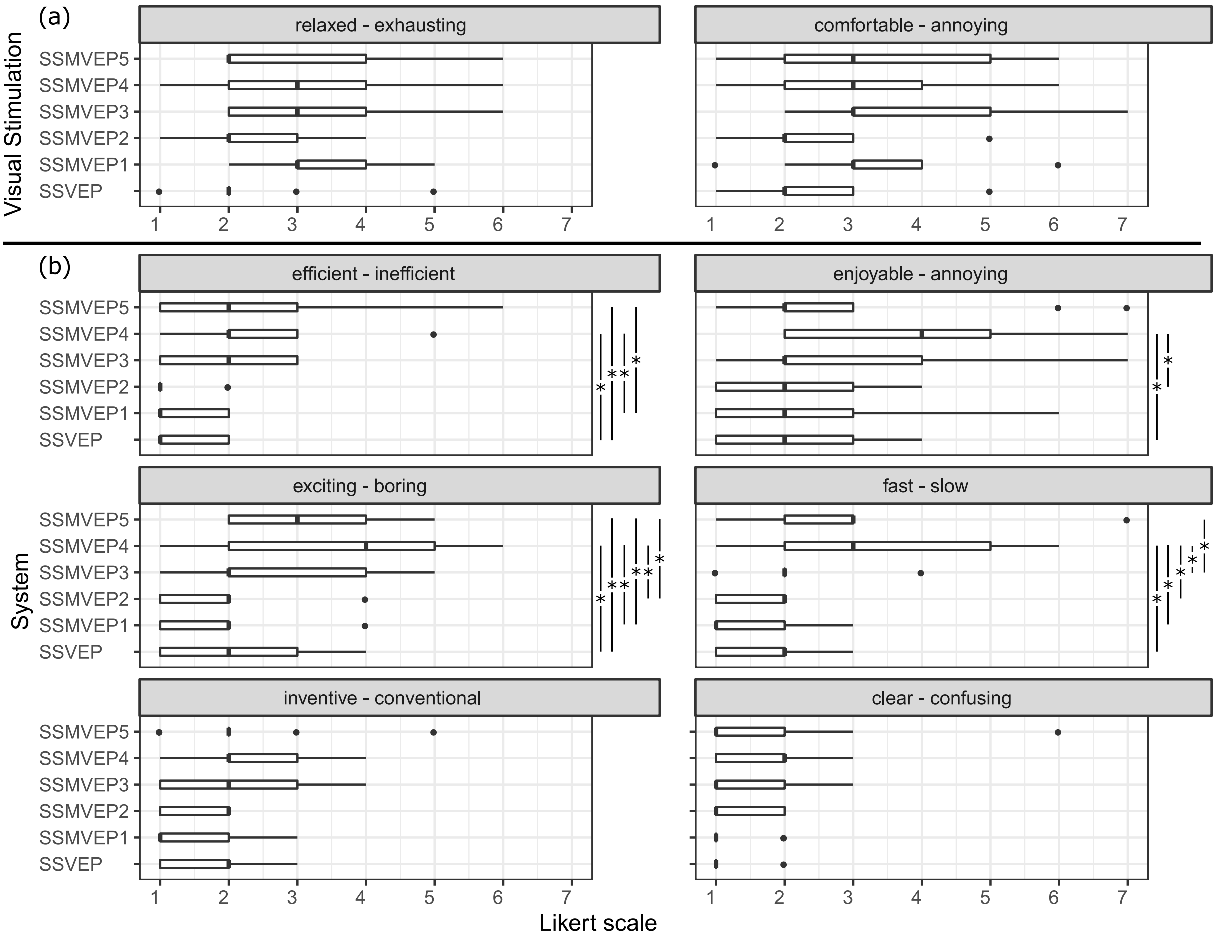

Figure S3: Participants' subjective responses to the questionnaire about the visual stimulation and the system. (**a**) These questions were asked after the training session (spelling of the word "BCI" with 9 trials), with a fixed classification window (4s). (**b**) These questions were asked after the main spelling task T_S (spelling of the word "INVITE" with 18-trial), with the classification time window varying between 1 and 16 seconds. The asterisks indicate statistical significance ($p < 0.05$) according to the pairwise comparison, utilizing the Wilcoxon signed-rank test (non-parametric t-test equivalent).

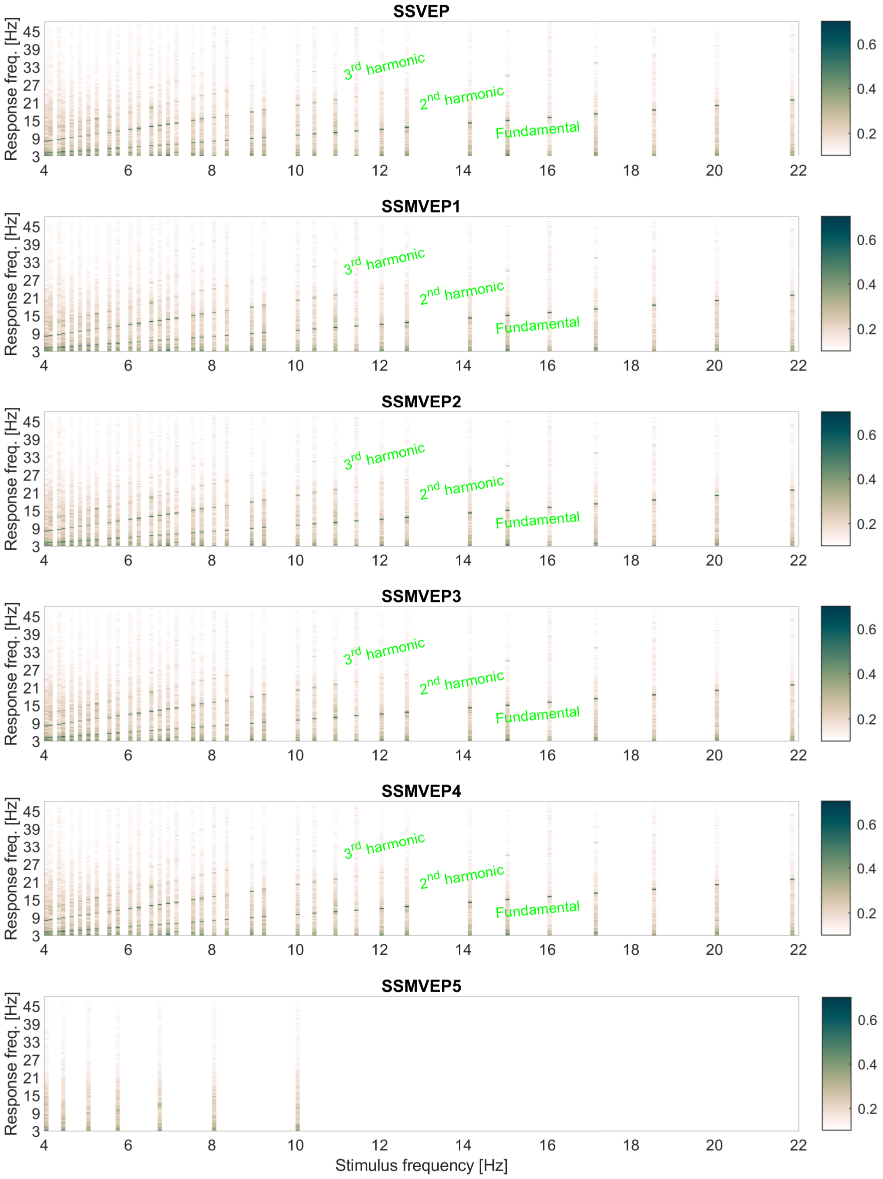

Figure S4: To better visualize the SSMVEP influence on the brain's visual area, we present an additional recording through the different SSMVEP designs. This figure shows the canonical correlation analysis (CCA) maximum correlation coefficient's (ρ) response, to the tested SSMVEP stimulus. The SSMVEP stimuli designs were presented in a single box (located at the center of the monitor with a small centered green fixation dot), for the following randomized frequencies: 4.00, 4.14, 4.29, 4.44, 4.62, 4.80, 5.00, 5.22, 5.45, 5.71, 6.00, 6.15, 6.32, 6.49, 6.67, 6.86, 7.06, 7.27, 7.50, 7.74, 8.00, 8.28, 8.57, 8.89, 9.23, 9.60, 10.00, 10.43, 10.91, 11.43, 12.00, 12.63, 13.33, 14.12, 15.00, 16.00, 17.14, 18.46, 20.00, and 21.82 Hz for 5 seconds, with a 5 seconds break; a sounds marked the beginning (1 second before) and the end of every trial. This whole recording of a single stimulus pattern (40 trails) took in total, 385 seconds. Between the stimulus patterns, the participant had a longer break, lasting 5 minutes, on average. The whole recording was limited to 1 hour. The participant received a financial reward for the participation. Due to time constrains, the last recording (SSMVEP5) was limited to the following frequencies: 4.00, 4.44, 5.00, 5.71, 6.67, 8.00, 10.00 Hz. The EEG for this spectrum analysis was recorded with 1200 Hz sampling rate utilising the same eleoctode location as in the main experiment. The ρ was calculated against a sine cosine matrix ranging from 3.00 to 48 Hz (with a $\Delta f = 0.01$ Hz) fundamental frequency only, for each trial.
